# Fragmentary Provings

**Published:** 1889-02

**Authors:** E. W. Berridge

**Affiliations:** London


### FRAGMENTARY PROVINGS.

E. W. BERRIDGE, M. D., LONDON.

1. *Murex Purpurea.*—1877, January 15th, Mrs. M. B. P. took two doses of 200th (Swan) under Dr. Swan's supervision.

Third day.—Felt very miserable, very fretful, irritable; no patience.

Fourth day.—On awaking had a swelled upper jaw—right side—as if there was an ulcerated gum, but there was no pain in it on pressure; accompanied with soreness of the median line under the nose, and also in right nostril; sensation as of an eyelash in right eye under upper lid—outer end. About four P. M. it became like a grain of sand, and was extremely painful; found a small ulcer in the lid about size of pin-head.

Fifth day.—Last night the pain extended to region of head, over and around the eye, with lachrymation; swelling under right angle of jaw.

Sixth day.—Eye better, face same, and about region of upper lip, right side is very sensitive to touch; submaxillary gland still swollen; stiffness in both legs, most in hip-joint, as if in the socket; stiff on first starting to walk; goes off on walking.

Seventh day.—On rising, nausea; nearly to vomiting, with great sickness all over. Relieved by breakfast. Face swells at night, and goes down during the day. Eye gets worse at night.

Eighth day.—In morning, despondent, low-spirited, irritable; better during the day. Urine flows very slowly, seems as if it would never stop dribbling.

Dr. Kent has made provings of Murex, which I hope he will publish.

2. *Culex Musca* (mosquito).—[Compare provings in *Transactions of I. H. A.*, 1886.]

Mrs. M. B. P. took about fifty pellets of 200 (Swan), two or three at a time, under Dr. Swan's direction; commenced September 7th, 1881. No symptoms for three weeks, then in afternoon felt hot all over, at the same time a sensation of an internal chill, which sometimes came in reality. Inclined to be hoarse; feels as if she had a cold, but has none; voice is rough, without reason; wakeful in early part of night.

September 26th.—Menses on time—afternoon—scanty at first; next day at eleven profuse, flow natural; pain in left groin in ovarian region; pain in back; flow mixed with mucus all through; fretting over anything which went wrong.

September 27th, 28th.—On the eve of a hysteria; headache in afternoon in forehead and right temple; blur before eyes and pain through them; aching in nape, relieved by lying flat on back without a pillow. Absolute sleeplessness till near morning.

3. *Gemiasma verdans* (*Salisbury*).—October 1st, Dr. S. Swan took three doses of 30th potency.

On October 7th and 8th, very sleepy in daytime. After breakfast on 8th, felt lassitude and heaviness in forehead; felt slightly chilly, as if it would not require much to bring on a chill. At noon had to take a nap, which continued till one P. M.; felt chilly all over, very slight; went to lunch, but had no appetite; no thirst. Began to grow warm all through; pulse 96, respiration 32; forehead felt hot inside; heat in face over malar bones; heat in eyes, and especially in lids; eyeballs hot and slightly congested; tongue slightly coated white, urine golden—sherry color—not very frequent. Stool after chilly feeling commenced, loose, lumpy, with large amount of flatus. During fever still sleepy; saliva has a metallic taste; taking hold of anything cold causes a chilly feeling. Dryness of throat, with slight soreness all round, as a ring below glottis, felt when swallowing, but the heat and dryness are felt all the time. Sound sleep from five to six P. M. No appetite for supper, but after a piece of toast and some grapes took a fair meal and felt much better afterward, though the heat continued.

4. *Lemon Juice.*—April 5th, Mrs. L. took 30th potency under Dr. Swan's direction.

April 6th.—During last night severe pains in chest woke her; could not take a long breath; it kept her awake about thirty minutes; relieved when sitting up.

April 18th.—All black before eyes; soon afterward sick at stomach. Dull ache in left arm and shoulder.

April 20th.—Left leg, from just above knee to ankle, ached so that it was impossible to keep quiet.

April 21st.—Pains in left leg the same, but only during evening. Short breath all day.

April 25th.—Short breath. Stitch in left side between ribs.

5. *Gallinæ stomachi tunica interior* (Ingluvin or fowl's gizzard).—1870, October 28th, Dr. Swan took one dose of 200th at five P. M.

October 29th.—Soreness of tongue, right side and edge. Hard, difficult stool, which is unusual. At ten A. M., took another dose of 200th. Increase of saliva; tingling of dorsum of tongue. Dryness and sense of heat in eyelids, without redness. Tongue indented by the teeth. At five P. M. repeated dose of 200th.

October 30th.—Dryness and stoppage of nose at night toward morning; dry scales in nose. Dry, hard stool, requiring straining to pass it. At ten A. M. took dose of 200th. At two P. M. soft stool. At five P. M. soft, lumpy stool with tenesmus, and the peculiar thrill and painful pressure on anus as in dysentery, and as if rectum would protrude; after stool, slight colicky pain in lower part of bowels; unpleasant sensation, slightly painful, in rectum sometime afterward.

6. *Liquorice.*—Dr. Swan took 6th potency. It caused fullness in nose as if he had taken cold; slight chilliness; pain in forehead, especially over right eye; sleepy.

7. *Poisoning by fish.*—Mrs. K., after eating scalded fish, especially mackerel. Redness and swelling of ears, nose, hands, elbows, knees, and shoulders, with intolerable itching. Dr. Swan cured her with one dose of *Scomber*$^{10m}$ (Swan).

8. *Poisoning by egg.*—A lady had intense cramps in stomach and umbilical region doubling her up with the most excruciating pain; relieved by brandy. Dr. Swan cured her with *Yelk of Egg*$^{10}$ (Swan).

9. *Gettysburg.*—In the *Hahnemannian Monthly*, VI, 389, Dr. Swan published a valuable proving of this mineral water, which has been clinically verified in spite of the *dictum* of a skeptic that it was a natural attack of rheumatic fever. Here is another verification sent me by Dr. Swan April 16th, 1882:

"A few days since I woke with a painfully stiff neck when I brought any strain on the left sterno-cleido-mastoideus or its attachment to the head; when quiet there was no pain. Took one dose of *Gettysburg*$^{mm}$ (Swan). Was entirely well on third day, but on second day had burning pains in pisiform bones of both hands."

---

In the report in the *Hahnemannian Monthly*, Vol. VI, the following corrections should be made:

Page 391, line twenty-five, for "*hands*" read "*hand.*"

" 391, line fifteen, after "*in*" read "*throat and posterior nares.*"

Page 393, line sixteen, for "*joints*" read "*spots.*"

" " line eighteen, after "*painful*" read "*on motion.*"

" 395, line four from bottom, for "*5th*" read "*7th.*"

